# Research on Processing Characteristics of *Sedum aizoon* L. and Development of Low-GI Meal Replacement Powder

**DOI:** 10.3390/foods15193379

**Published:** 2026-09-23

**Authors:** Ying Wang, Ruohan Huang, Mengxiao Li, Yishi Guo, Zihan Zhao, Jianmei Mao, Wanzhong He, Qilin Zhuoma, Juxiu Li, Min Zhang

**Affiliations:** 1 College of Food Science and Engineering, Northwest A&F University, Yangling, Xianyang 712100, China; 2 Productivity Promotion Center of Diqing Tibetan Autonomous Prefecture, Shangri-La 674499, China

**Keywords:** *Sedum aizoon* L., vegetable powder, processing characteristics, low-GI, meal replacement powder

## Abstract

Low Glycemic Index (low-GI) meal replacement powders are increasingly sought for weight management. *Sedum aizoon* L. is a promising vegetable raw material, yet its processing characteristics remain poorly characterized. This study systematically evaluated the effects of four pretreatments (untreated, freeze–thaw, blanching, blanching plus green protection) and two drying methods (hot-air drying, freeze drying) on *S. aizoon* L. from the Diqing Plateau, *Sedum aizoon* L., and optimized a low-GI meal replacement formula. The results revealed that blanching and blanching plus green protection shortened hot-air-drying time by approximately 50% and improved the flowability and compactness of powder (Hausner ratio 1.26, Carr index 20.46%). Qualitative and quantitative analyses revealed that *Sedum aizoon* L. contained various bioactive substances such as flavonoids and phenolic acids. Freeze drying better retained polyphenols, flavonoids, and vitamin C; the freeze–thaw plus freeze drying exhibited the optimal color protection effect with a chlorophyll content of 1.4843 mg/g. Dried *Sedum aizoon* L. was evaluated by a trained panel plus electronic nose analysis, while the meal replacement powder was assessed by electronic nose and electronic tongue. A single-factor test, a Plackett–Burman test and a response surface methodology were adopted to optimize the optimal formula of meal replacement powder: *Sedum aizoon* L. powder 2.3 g, skim milk powder 2.8 g, sugar powder 1.6 g, grain powder 4 g, nut powder 2.6 g, and konjac powder 0.55 g. The meal replacement powder has low estimated GI (eGI) value (eGI < 55) and high dietary fiber content (43.4 g/100 g), with superior aroma and taste versus a commercial reference. These findings support the industrial production of *Sedum aizoon* L.-based low-GI meal replacement food.

## 1. Introduction

According to the 2025 World Obesity Map released by the World Obesity Federation, the proportion of overweight and obese adults worldwide is projected to rise to 50% by 2030. Consumer demand for diets supporting weight loss, glycemic control and stable blood glucose levels keeps growing, and convenient meal replacement foods with low GI, high fiber and high functionality have dominated the market. Low-GI foods supply energy while stabilizing human blood glucose [1], and have attracted wide attention for enhancing satiety and supporting weight management [2,3]. Meal replacement powders can provide balanced nutrition via raw material compounding [4], and exert effects such as weight loss, blood lipid regulation and blood glucose control [5,6,7], leading to continuous expansion of the market scale. Most commercial meal replacement powders are made of cereals and legumes [8,9], with drawbacks including monotonous nutritional composition, severe product homogenization and insufficient functional properties [10]. Vegetable-based meal replacement powders make up for the deficiencies of traditional meal replacement powders by virtue of natural low-GI characteristics and abundant bioactive nutrients, and represent an important direction for innovation and upgrading of the meal replacement industry. Therefore, screening highly compatible vegetable raw materials and optimizing compound formulas are the core directions for developing vegetable-based low-GI meal replacement powders.

*Sedum aizoon* L., a perennial herb belonging to the genus Phedimus of Crassulaceae under Rosales, is rich in bioactive components including polyphenols, polysaccharides, sterols and flavonoids [11,12], and exhibits favorable antioxidant, anti-inflammatory, antibacterial and metabolism-regulating activities [13,14,15,16]. Among them, *Sedum aizoon* L. cultivated in the plateau region of Diqing Tibetan Autonomous Prefecture, Yunnan Province, China exhibits superior natural quality owing to unique ecological conditions including high altitude, pollution-free environment and mineral-rich soil. It serves as a distinctive high-quality raw material for developing functional meal replacements, with abundant planting resources and enormous development potential. Nevertheless, fresh *Sedum aizoon* L. has high water content and vigorous postharvest respiratory metabolism. It is susceptible to microbial infection and rot, with a short natural storage life [17], and its current development and utilization forms are relatively limited. Existing studies mostly focus on extraction process optimization and functional activity verification of bioactive components in *Sedum aizoon* L. [18,19]. There is a lack of systematic research on its processing adaptability, and the influence of key pretreatment and processing technologies on its quality remain unclear [20,21]. The absence of standardized parameters for industrial production restricts the high-quality development of the *Sedum aizoon* L. industry.

While *S. aizoon* L. is known to be rich in polyphenols, flavonoids, and polysaccharides, the bioavailability and intestinal absorption of these constituents have not been systematically characterized. It is well established that thermal processing and drying operations can alter the cell wall integrity, extractability, and structural stability of phytochemicals, thereby influencing their bioaccessibility during gastrointestinal digestion and subsequent absorption [22]. Therefore, understanding how pretreatment and drying methods affect the contents of bioactive components is critical for evaluating the nutritional value of processed *Sedum aizoon* L. products.

In view of the above research progress, plateau-grown *Sedum aizoon* L. from Diqing Tibetan Autonomous Prefecture, Yunnan Province, China, was used as raw material. Four pretreatments (untreated, freeze–thaw, boiling-water blanching, color protection combined with boiling-water blanching) and two drying methods (hot-air drying and vacuum freeze drying) were adopted. The variations in drying characteristics, powder properties, nutritional characteristics, sensory attributes and antioxidant capacity of dried *Sedum aizoon* L. products were systematically investigated to clarify the effects of different processing protocols on the overall quality, filling the research gap regarding the processing characteristics of *Sedum aizoon* L. Furthermore, single-factor experiments combined with response surface methodology were carried out to optimize the formulation of meal replacement powder. A low-GI meal replacement powder of *Sedum aizoon* L. with desirable nutritional functions and acceptable sensory quality was developed. This study provides theoretical references and technical support for the high-value utilization of plateau-grown *Sedum aizoon* L., as well as the industrial production and commercial application of its low-GI meal replacement powder.

## 2. Materials and Methods

### 2.1. Materials and Instruments

*Sedum aizoon* L., black highland barley, and quinoa were obtained from the Diqing Tibetan Autonomous Prefecture, Yunnan Province. Oat flakes, walnut powder, cooked peanut powder, skim milk powder, white granulated sugar, xylitol, konjac powder were purchased from commercial suppliers.

Petroleum ether, ethanol, chloroform, sodium hydroxide and sulfuric acid, all of an analytical grade, were purchased from Xilong Scientific Co., Ltd. (Chengdu, China). Glacial acetic acid, and sodium thiosulfate, all of an analytical grade, were obtained from Chengdu Kelong Chemical Reagent Co., Ltd. (Chengdu, China). Copper sulfate, potassium sulfate, and sodium acetate, all of an analytical grade, were purchased from Guangdong Guanghua Sci-Tech Co., Ltd. (Shantou, China). Potassium iodide was obtained from Sinopharm Chemical Reagent Co., Ltd. (Shanghai, China) Pancreatic α-amylase, amyloglucosidase, DPPH, and ABTS were purchased from Shanghai Yuanye Bio-Technology Co., Ltd. (Shanghai, China).

The following instruments were used: GL124-1SCN electronic balance (Sartorius Scientific Instruments Co., Ltd., Beijing, China); HH-2 water bath (Changzhou Yuexin Instrument Manufacturing Co., Ltd., Changzhou, China); 101-00B hot air-drying oven (Shaoxing Supo Instrument Co., Ltd., Shaoxing, China); UV-1900i UV-Vis spectrophotometer (Shimadzu Instruments (Suzhou) Co., Ltd., Suzhou, China); TA.XT PLUS/50 texture analyzer (Stable Micro Systems (SMS), Schwerin, UK).

### 2.2. Experimental Methods

#### 2.2.1. Pretreatment and Drying Technology of *Sedum aizoon* L.

After fresh *Sedum aizoon* L. was cleaned and drained, four pretreatment groups were set: untreated, freeze–thaw, blanching in boiling water, and color protection combined with boiling water blanching. For freeze–thaw pretreatment, samples were frozen at −5 °C for 48 h and then thawed at room temperature. For boiling water blanching, samples were blanched in boiling water for 60 s at a solid–liquid ratio of 1:20 (g:mL), followed by rapid cooling with cold water after taking out. Color protection treatment was carried out by blanching samples in boiling zinc acetate solution with a mass fraction of 0.05%. After draining, samples were dehydrated via hot air drying and freeze drying, respectively. Hot air drying was performed at 60 °C until constant weight was achieved. For freeze drying, samples were pre-frozen at −80 °C for 8 h, then dried to constant weight with a vacuum freeze dryer under the conditions of cold trap temperature of −80 °C and vacuum degree less than 10.0 Pa.

#### 2.2.2. Determination of Drying Characteristics

During drying, intermittent weighing was adopted to monitor the mass change of samples, and the moisture ratio (MR) was calculated according to Equation (1):

(1) $$MR=\frac{M_{t}-M_{e}}{M_{0}-M_{e}}$$

where MR represents the moisture ratio of *Sedum aizoon* L., unit: g/100 g; M_0_, M_e_ and M_t_ are the dry basis moisture content at initial state, equilibrium state and time t, respectively, unit: g/g.

#### 2.2.3. Preparation of *Sedum aizoon* L. Powder

The preparation process of *Sedum aizoon* L. powder consisted of cleaning and cutting, alkali soaking, blanching for green protection, drying, and pulverization. Cleaning and cutting: Fresh green *Sedum aizoon* L. with uniform size and no mechanical damage was selected, cleaned, and cut into segments of 3–5 cm in length. Alkali soaking: The soaking procedure of *Sedum aizoon* L. was performed according to the method reported by Liu Yang [23]. The samples were immersed in 0.15% anhydrous sodium carbonate solution with a solid–liquid ratio of 1:15 for 30 min. Blanching for green protection: The soaked *Sedum aizoon* L. was taken out and blanched in a water bath containing zinc acetate as green-protecting agent. Afterwards, the samples were cooled with cold running water for 15 min and drained thoroughly. Pulverization: All dried *Sedum aizoon* L. samples were pulverized by an ultrafine pulverizer and sieved through a 100-mesh sieve.

#### 2.2.4. Determination of Powder Properties

Dried *Sedum aizoon* L. was pulverized with a high-speed grinder and sieved through a 50-mesh screen. Bulk density was determined in accordance with GB/T 20316.2-2006 [24]. The second part of the determination of the bulk density of abrasive grains involved micro-powders; tapped density was measured following GB/T 21354-2008 [25]. Regarding powder products, the general method for the determination of tapped density was as follows. The Hausner ratio was calculated by Equation (2), and Carr’s index was calculated by Equation (3), as shown below:

(2) $$\mathrm{Hausner}\;\mathrm{ratio}/(\%)=\frac{\rho_{t}}{\rho_{0}}$$

(3) $$\mathrm{Carr}’s\;\mathrm{index}/(\%)=\frac{(\rho_{t}-\rho_{b})\times 100}{\rho_{b}}$$

where ρ_t_ is the tapped density of powder, in g/mL; ρ_b_ is the bulk density of powder, in g/mL.

#### 2.2.5. Determination of Nutritional Characteristics

##### Qualitative Analysis of Components in *Sedum aizoon* L.

After pulverizing *Sedum aizoon* L., 0.5 g of the powder was weighed and extracted with 25 mL of 80% ethanol under ultrasonic treatment for 40 min, followed by water bath extraction at 60 °C for 2 h. The mixture was then centrifuged at 4000 r/min for 15 min, and the supernatant was collected and stored at 4 °C. The ethanol extract of *Sedum aizoon* L. was analyzed by high-resolution ion mobility liquid chromatography–mass spectrometry. The chromatographic conditions referred to the research method reported by He Jiali et al. [26]. The structures of components in the extract were analyzed combined with mass spectrometry data from the traditional Chinese medicine natural product database.

Chromatographic conditions: column temperature 40 °C; mobile phase: mobile phase A was acetonitrile containing 0.1% formic acid, mobile phase B was aqueous solution containing 0.1% formic acid; gradient elution procedure: 0–2 min (5% A), 2–42 min (5–95% A), 42–47 min (95% A), 47.1–50 min (5% A); flow rate 0.3 mL/min; injection volume 5 μL. Mass spectrometry conditions: heated electrospray ionization (HESI-II) ion source; simultaneous acquisition in positive and negative ion modes; mass scan range (*m*/*z*): 100–1500; spray voltage: 3.0 kV (positive mode)/2.5 kV (negative mode); capillary temperature 320 °C, probe heater temperature 350 °C; scan mode: Full MS-ddMS2.

##### Quantitative Analysis of Components in *Sedum aizoon* L.

The content of vitamin C was determined by the 2,6-dichloroindophenol titration method specified in GB 5009.86-2025 [27] National Food Safety Standard—Determination of Ascorbic Acid in Foods. Total polyphenols were quantified via the Folin–Ciocalteu method, total flavonoids by the NaNO_2_-AlCl_3_-NaOH colorimetric method, and total polysaccharides using the phenol–sulfuric acid method.

#### 2.2.6. Determination of Antioxidant Activity

Determination of DPPH radical scavenging capacity: The method reported by Liu et al. [28] was slightly modified. Briefly, 4 mL of 0.2 mmol/L DPPH ethanol solution was mixed with 0.5 mL *Sedum aizoon* L. extract, and the mixture was incubated in the dark at room temperature for 30 min. The absorbance was determined by an ultraviolet spectrophotometer at 517 nm, with ethanol used instead of the extract as blank control. The DPPH radical scavenging rate was calculated according to Equation (4).

(4) $$I_{DPPH}=1-\left(\frac{A_{1}-A_{2}}{A_{0}}\right)\times 100\%$$

where A_0_ refers to the absorbance of absolute ethanol at 517 nm; A_1_ represents the absorbance of the test solution at 517 nm; A_2_ is the absorbance of the blank sample at 517 nm.

Determination of ABTS radical scavenging capacity: 200 μL ABTS solution was mixed with 200 μL potassium persulfate solution and kept in the dark at room temperature for 16 h. The ABTS working solution was then diluted with absolute ethanol until its absorbance reached approximately 0.700. Afterwards, 0.5 mL sample solution was mixed with 6 mL ABTS working solution, and the absorbance was measured with an ultraviolet spectrophotometer at 734 nm.

(5) $$I_{ABTS}=\left(\frac{A_{4}-A_{5}}{A_{3}}\right)\times 100\%$$

where A_3_ is the absorbance of absolute ethanol at 734 nm; A_4_ denotes the absorbance of test solution at 734 nm; A_5_ stands for the absorbance of blank sample at 734 nm.

#### 2.2.7. Determination of Sensory Properties of *Sedum aizoon* L.

##### Color

The color of *Sedum aizoon* L. was measured by a colorimeter, and L*, a*, b* values were calculated in accordance with the GB/T 7621-2008 [29] uniform color space and color difference formula.

##### Determination of Chlorophyll Content

The pretreated *Sedum aizoon* L. was pulverized, and a 0.5 g sample was weighed, followed by adding 10 mL ethanol–acetone extractant (*V/V* = 1:1). The mixture was sealed, shaken well, and extracted in the dark at room temperature for 5 h. After filtration, the absorbance of the filtrate was determined at 645 nm and 663 nm, respectively.

(6) $$w=(8.05A_{1}+20.29A_{2})\times V/(1000\;m)$$

where w is the total chlorophyll content, unit: mg/g; A_1_ refers to the absorbance of test solution at 663 nm; A_2_ represents the absorbance of test solution at 645 nm; V is the volume of test solution, unit: mL; m is the mass of sample, unit: g.

##### Volatile Flavor Profiling by Electronic Nose

Flavor substances were detected by an electronic nose (PEN3, Airsense Analytics, Schwerin, Germany), and the corresponding substance types of the ten sensors are shown in Table 1. Each sample (2.0 g) was placed in a 40 mL sealed vial and equilibrated at 25 °C for 10 min before measurement; the detection time was 120 s with a flush time of 120 s.

##### Human Sensory Evaluation

Fifteen trained professional sensory panelists (8 females and 7 males, aged 22–45 years) participated. All panelists completed a training program comprising descriptor development, reference standard recognition, and scale anchoring exercises prior to formal evaluation. Panelists evaluated eight groups of dried *Sedum aizoon* L. samples from five dimensions: grassy odor, roasted aroma, bitterness, sourness, and earthy odor. A 10-point scale (0–9) with an interval of 1 was adopted: 0 = undetectable, 1 = faint, 5 = distinct, 9 = extremely intense. All samples were coded with random three-digit numbers and presented in a randomized order under white fluorescent lighting in individual sensory booths; filtered water was provided for palate cleansing between samples. The average score of each dimension was calculated.

#### 2.2.8. Formula Design of Meal Replacement Powder

##### Single-Factor Experiment

On the basis of preliminary experimental results, single-factor experiments were carried out to investigate the effects of raw materials on the color, flavor, reconstitution property, taste and mouthfeel of meal replacement powder. The experimental gradients were set as follows: *Sedum aizoon* L. powder addition of 1.0, 2.0, 3.0, 4.0, 5.0 g; compound grain powder addition of 3.0, 4.0, 5.0, 6.0, 7.0 g; compound nut powder addition of 1.0, 1.8, 2.6, 3.4, 4.2 g; skim milk powder addition of 1.0, 1.5, 2.0, 2.5, 3.0 g; compound sugar powder addition of 0.5, 1.0, 1.5, 2.0, 2.5 g. The optimal addition range of each raw material was determined, and the sensory evaluation criteria are shown in Table 2. Specifically, the compound grain powder was prepared by mixing black highland barley powder, quinoa powder and oat flakes at a mass ratio of 2:3:3. The compound nut powder was obtained by blending walnut powder and roasted peanut powder at a mass ratio of 1:1, while the compound sugar powder was compounded with white granulated sugar and xylitol at a mass ratio of 1:1.

##### Plackett–Burman Design

Based on single-factor experimental results, sensory score was used as the indicator to screen main effect factors of the five raw materials and analyze their significance. The factor-level table is shown in Table 3.

##### Box–Behnken Design

According to the results of single-factor and Plackett–Burman experiments, sensory score was taken as evaluation index. Response surface optimization and analysis were further carried out on the effects of *Sedum aizoon* L. powder (A), skim milk powder (B) and sugar powder (C) on sensory scores of meal replacement powder. The design of factor levels is listed in Table 4.

##### Consistency Assessment by Texture Analysis

Meal replacement powders must maintain a homogeneous suspension after reconstitution; excessive stratification or sedimentation impairs mouthfeel and consumer acceptance. Therefore, consistency and stability after reconstitution were considered alongside sensory attributes during formula optimization, and konjac powder was evaluated as a thickening and stabilizing agent. The optimal dosage of konjac flour was determined based on the viscosity of the product. The meal replacement powder was mixed with hot water at a solid–liquid ratio of 1:10 and stirred evenly. After standing for 5 min, texture analysis was performed at 25 °C. The maximum negative peak force (g) at the second compression of the probe was recorded as the result.

##### Electronic Nose and Electronic Tongue Analyses

Electronic nose: The volatile flavor profile of the meal replacement powder was analyzed using an electronic nose (PEN3, Airsense Analytics, Germany). Each sample (2.0 g) was reconstituted with 80 °C water at a solid-to-liquid ratio of 1:10 (*w*/*v*), stirred evenly, and equilibrated at 25 °C for 10 min in a sealed vial before measurement.

Electronic tongue: The taste profile was objectively characterized using an electronic tongue (SA402B, Insent Inc., Atsugi, Japan). Each sample was reconstituted with 80 °C water at 1:10 (*w*/*v*), stirred evenly, and centrifuged at 3000 r/min for 10 min; the supernatant was used for measurement. The sensors were calibrated and equilibrated according to the manufacturer’s protocol, and taste attributes including sourness, bitterness, astringency, saltiness, umami, and their aftertastes were recorded. A commercial vegetable-based meal replacement powder (containing kale powder, oat flakes, and flaxseed) was analyzed in parallel as a reference. Each measurement was performed in triplicate.

#### 2.2.9. Determination of Physicochemical Indexes of Meal Replacement Powder

Dispersibility: Weigh 2 g of meal replacement powder and spread it evenly on the surface of 100 mL water. Record the time taken for the powder to fully disperse under static standing and magnetic stirring, respectively, which is defined as dispersion time.

Solubility: 5.0 g meal replacement powder and 30 mL ultrapure water were added into a 100 mL beaker, followed by magnetic stirring at 1000 r·min^−1^ for 30 min. The mixture was transferred to a 50 mL volumetric flask and made up to volume with ultrapure water. A 10 mL aliquot of the constant-volume solution was centrifuged at 3000 r·min^−1^ for 10 min. The supernatant was transferred to a beaker, dried in an oven at 105 °C, and the solubility was calculated.

#### 2.2.10. Determination of Nutritional Indexes of Meal Replacement Powder

Moisture content was determined by a direct drying method in accordance with national standard GB 5009.3-2016 [30]; fat content was measured by Soxhlet extraction method per GB 5009.6-2016 [31]; protein content was tested via Kjeldahl method following GB 5009.5-2025 [32]; dietary fiber content was determined according to GB 5009.88-2023 [33].

Determination of GI value: Weigh 0.20 g meal replacement powder sample, add 15 mL sodium acetate buffer (pH 5.2, 0.20 mol/L), 10 mL porcine pancreatic α-amylase (300 U/mL) and glucoamylase (15 U/mL). Shake the mixture in a thermostatic water bath at 37 °C (150 r/min) for 0, 30, 60, 90, 120 and 180 min, respectively. Collect 2 mL hydrolysate at each time point, inactivate enzymes in boiling water bath and centrifuge. Take 100 μL supernatant, dilute to 1 mL with distilled water, and measure absorbance at 540 nm. The starch hydrolysis curve was plotted based on hydrolysis time and starch hydrolysis rate. The area under the hydrolysis curve (AUC) of each sample within 0–180 min was calculated and denoted as AUC_Sample_. White bread was used as the reference food with AUC recorded as AUC_Reference_. The starch hydrolysis index (HI) of the sample was determined, and the glycemic index (GI) was further calculated with the formula shown below:

(7) $$HI=\frac{{AUC}_{Sample}}{{AUC}_{Renferce}}\times 100\%$$

(8) GI=39.71+0.549×HI

### 2.3. Data Processing

All experiments were performed in triplicate, and results are expressed as mean ± standard deviation. Data were analyzed by one-way ANOVA using SPSS 22.0, with *p* < 0.05 indicating significant difference. Graphs were plotted with Origin 2022, and data sorting and chart preparation were conducted in Excel.

## 3. Results

### 3.1. Analysis of Drying Characteristics

This study explored the effects of different pretreatments (untreated, freeze–thaw, blanching, blanching combined with green preservation) on the drying characteristics of *Sedum aizoon* L. Owing to the continuous-operation requirement of the vacuum-freeze-dryer, intermediate weighing was not possible during the freeze-drying runs; only the initial and final masses were recorded for those samples. Therefore, Figure 1 presents the drying curves solely for the hot-air-drying groups, in which samples were weighed at 3 h intervals without interrupting the drying process. The moisture ratio of *Sedum aizoon* L. (MR) is defined as the ratio of material moisture content at a given drying time to the initial moisture content. An MR value closer to 0 indicates more sufficient drying. The variations in moisture ratio of *Sedum aizoon* L. with different pretreatments are shown in Figure 1.

It can be seen from Figure 1 that all drying curves conform to the typical drying characteristic curves of food, and the MR values of the four sample groups decrease with the extension of drying time. In terms of differences among treatment groups, the control group (without pretreatment) exhibited a significantly slower decline rate in terms of moisture ratio (MR) than the other groups. Taking the time required to reach an MR of 0.1 as the comparison index, the drying times of the blanching group, the blanching combined with green-preservation group, and the freeze–thaw group wrtr reduced by 52.00%, 49.33% and 28.00%, respectively, compared with the control group, indicating that all three pretreatments could shorten drying duration. The blanching group and blanching with green-preservation group exhibited the shortest drying time. The approximately 50% reduction in hot-air-drying time induced by blanching is consistent with previous reports on leafy vegetables [34]. Boiling-water blanching removes the waxy cuticle on the surface of *Sedum aizoon* L., loosens cellular structures, and unblocks moisture diffusion pathways. Freeze–thaw treatment disrupts native cell architecture, releases intracellular water, and improves cell wall permeability. It lowers the resistance to moisture migration [35] during drying and thereby enhances drying efficiency. In contrast, the untreated control group maintained intact cellular structures, requiring moisture to gradually migrate from the inner tissue to the surface, which prolonged drying duration.

In summary, blanching and blanching plus green protection were the most effective pretreatments for shortening hot-air-drying time.

### 3.2. Analysis of Powder Properties

Besides drying characteristics, powder properties are also key indicators for evaluating the processing adaptability and application performance of vegetable powder. The compactness and flowability of powder are comprehensively assessed via bulk density, tapped density, Hausner ratio and Carr index. Bulk density represents the density of powder under natural stacking, while tapped density reflects the compactness of powder after tapping. Hausner ratio and Carr index can characterize the compressibility and cohesiveness of powder. A Hausner ratio closer to 1 and a lower Carr index indicate better powder flowability [36]. As shown in Table 5, the hot-air-drying group (-r) had significantly higher bulk density and tapped density than the freeze-drying group (-d), and its overall powder flowability was superior to that of the freeze-drying group. This can be attributed to the fact that hot-air drying shrinks and compacts materials, which facilitates powder flow. In contrast, freeze drying produces a porous and loose structure, resulting in lower powder density and poorer flowability. In addition, different pretreatments exerted more remarkable effects on the density and flowability of samples subjected to hot-air drying. The bulk density and tapped density of the blanching–green preservation group (Group 3-r) were higher than those of other pretreatments (Groups 0-r, 1-r and 2-r), with a Hausner ratio of 1.26 and a Carr index of 20.46%. The results indicated that blanching combined with green preservation could improve the compactness and flowability of powder after hot-air drying. By contrast, freeze–thaw treatment (Group 1-r) showed weaker improvement in flowability compared with the blanching group and blanching–green preservation group.

Overall, the blanching-plus-green-protection/hot-air-drying combination yielded the most compact and flowable powder.

### 3.3. Analysis of Nutritional Characteristics

#### 3.3.1. Qualitative Analysis of Components in *Sedum aizoon* L.

The composition of bioactive compounds in *Sedum aizoon* L. is susceptible to environmental conditions of the producing area, and raw materials from different regions exhibit obvious compositional differences. In this study, *Sedum aizoon* L. produced in Diqing Tibetan Autonomous Prefecture, Yunnan Province, was selected as the raw material. Since systematic research on its bioactive components from this origin has not yet been reported, high-performance liquid chromatography–mass spectrometry (HPLC-MS) was first adopted to qualitatively identify the constituents in the ethanolic extract of *Sedum aizoon* L. in order to clarify its chemical basis. HPLC-MS analysis identified a complex profile of bioactive constituents in *S. aizoon* L. from Diqing, including flavonoids (e.g., rutin, quercetin, luteolin, kaempferol), phenolic acids (e.g., caffeic acid, p-coumaric acid), triterpenoids (e.g., ursolic acid, oleanolic acid), fatty acids, vitamins (C, K_1_), organic acids, and anthraquinones. The complete list of identified compounds with molecular formulas and response values is provided in Supplementary Table S1. 

#### 3.3.2. Quantitative Analysis of Components in *Sedum aizoon* L.

*Sedum aizoon* L. contains a variety of bioactive substances and possesses high nutritional value, whereas the contents of these compounds are susceptible to pretreatments and drying methods. Among different drying techniques, the contents of total polyphenols, total flavonoids, polysaccharides and vitamin C in hot-air-dried *Sedum aizoon* L. were generally lower than those in vacuum-freeze-dried samples. The high temperature during hot-air drying tends to induce oxidation or degradation of heat-sensitive polyphenols and flavonoids, leading to losses of bioactive components [37]. This finding is consistent with the conclusion reported by Zhu et al. [38] that vacuum freeze drying retains significantly higher contents of bioactive compounds than thermal drying.

Polyphenols and flavonoids are vital nutritional and bioactive constituents of *Sedum aizoon* L., and their contents are significantly affected by pretreatment procedures. The variations in their contents under different pretreatment combinations are presented in Figure 2a,b. The order of total polyphenol content was control group > freeze–thaw group > blanching group > blanching–green preservation group. Freeze–thaw treatment exerted a weak destructive effect on polyphenols, and no significant reduction in polyphenol content was observed compared with the control group. By contrast, blanching and blanching combined with green preservation accelerated the oxidation and leaching of polyphenols under high temperature, resulting in a significant decrease in polyphenol content. The variation trend of total flavonoid content was roughly consistent with that of total polyphenols. Notably, the total flavonoid content of freeze–thaw samples subjected to vacuum freeze drying was even significantly higher than that of the control group. This phenomenon may be attributed to cell structure disruption induced by freeze–thaw treatment, which facilitates the leaching and release of flavonoids [39]. After blanching, the waxy layer on the surface of *Sedum aizoon* L. was destroyed, and high-temperature drying accelerated the oxidation and loss of flavonoids in leaves.

Total polysaccharides are nutritional and functional components in *Sedum aizoon* L., and analyzing their content variations can further elucidate the changing rules of bioactive components during processing. Figure 2c shows the total polysaccharide contents of *Sedum aizoon* L. under different processing combinations. Various pretreatments exerted varying degrees of effects on total polysaccharide content. Compared with the control group, freeze–thaw, blanching, and blanching–green preservation treatments significantly increased total polysaccharide content. This trend agrees with the finding reported by Song [40] that color-protective blanching yielded significantly higher total flavonoid content in Artemisia selengensis than the untreated group. Among all treatments, blanching exhibited the best retention of polysaccharides. A possible explanation is that the high temperature during blanching caused limited structural damage to polysaccharides and thus avoided remarkable polysaccharide loss.

In summary, compared with vacuum freeze drying, hot-air drying reduced the contents of bioactive substances to some extent. Under the same drying method, freeze–thaw pretreatment caused no significant reduction in polyphenol and flavonoid contents compared with the untreated control, whereas blanching and blanching combined with green preservation facilitated better retention of total polysaccharides and vitamin C.

In summary, compared with vacuum freeze drying, hot-air drying reduces the contents of bioactive substances in *Sedum aizoon* L. to a certain extent. Under the same drying method, freeze–thaw pretreatment increases the contents of polyphenols and flavonoids, while blanching and blanching combined with green preservation facilitate better retention of total polysaccharides and VC.

Taken together, freeze drying better preserved heat-sensitive bioactive compounds, whereas blanching-assisted hot-air drying better retained vitamin C and polysaccharides.

### 3.4. Analysis of Antioxidant Activity

In this study, the in vitro antioxidant capacity of *Sedum aizoon* L. under different pretreatments and drying methods was further characterized by measuring DPPH· radical scavenging activity and ABTS^+^ radical scavenging activity, and the results are displayed in Figure 3. DPPH· radical scavenging capacity and ABTS^+^ radical scavenging capacity are common evaluation indicators for measuring the antioxidant activity of samples; higher scavenging rates correspond to stronger antioxidant capacity. As illustrated in the figure, the influence of drying methods on ABTS^+^ and DPPH· radical scavenging capacities was consistent with that on polyphenols and flavonoids, indicating that hot-air drying caused severe loss of antioxidant components in all pretreatment groups. Under different pretreatments, the ABTS^+^ and DPPH· radical scavenging capacities of *Sedum aizoon* L. increased in the freeze–thaw group but decreased in the blanching group and blanching with green preservation group. This trend was basically consistent with the variation of total polyphenols, total flavonoids and VC contents, demonstrating that the loss of antioxidant components directly weakened the scavenging capacities against ABTS^+^ and DPPH· radicals. The ABTS^+^ and DPPH· radical scavenging capacities of *Sedum aizoon* L. were largely correlated with the contents of polyphenols and flavonoids [41]. In addition, VC is a water-soluble antioxidant with strong reducibility, which can not only scavenge free radicals directly, but also synergistically boost the antioxidant effects of polyphenols and flavonoids. Although freeze–thaw treatment leads to partial VC loss, it imposes slight damage on polyphenols, flavonoids and other components. Meanwhile, cell structure destruction induced by freeze–thaw facilitates the release of these bioactive substances, thereby resulting in relatively high antioxidant activity.

Thus, antioxidant activity was closely associated with polyphenol and flavonoid contents across all processing combinations.

### 3.5. Analysis of Sensory Characteristics

#### 3.5.1. Color Evaluation of *Sedum aizoon* L.

The effects of different processing combinations on the color of *Sedum aizoon* L. are shown in Table 6. L* stands for the lightness of samples. Freeze-dried groups (marked with -d) generally possessed higher L* values, among which the freeze–thaw combined with freeze-drying group (1-d group) showed the highest L* value of 61.99 ± 1.00. By contrast, hot-air-dried groups (marked with -r) exhibited lower L* values, and the lightness order was 0-r group > 1-r group > 3-r group > 2-r group. This observation was highly consistent with the finding that blanching triggered the loss of polyphenols and flavonoids. Moreover, the addition of color-protecting agents could effectively mitigate the adverse impacts of blanching on the color of *Sedum aizoon* L. Positive a* values represent redness and negative values represent greenness. Distinct green tones were retained in freeze-dried *Sedum aizoon* L., whereas all hot-air dried treatment groups showed faded and darkened green with no significant intergroup differences (*p* > 0.05), and even a reddish tendency. This was related to the variation in chlorophyll content after drying. Prolonged high-temperature drying causes chlorophyll degradation and demagnesification to form pyropheophytin [42], which ultimately leads to browning. In addition, the b* values of all treatment groups were positive, indicating an overall yellowish surface color, which was associated with chlorophyll degradation and oxidative browning of *Sedum aizoon* L. Different pretreatments exerted slight effects on the b* values of freeze-dried samples, while more obvious changes occurred after hot-air drying, with the order of 0-r group > 3-r group > 1-r group > 2-r group. This further demonstrated that green preservation treatment could largely maintain the original color of *Sedum aizoon* L. and alleviate color deterioration induced by hot-air drying.

Chlorophyll is the main pigment of *Sedum aizoon* L., and its content variation can directly explain the differences in color parameters. Therefore, this study further investigated the effects of different processing techniques on chlorophyll content of *Sedum aizoon* L., and the results are presented in Table 7. All freeze-dried groups (-d groups) had significantly higher chlorophyll contents than hot-air-dried groups (-r groups), which indicated that high temperature during hot-air drying accelerated chlorophyll decomposition, resulting in higher redness a* values and darker color. The effect of pretreatments on chlorophyll content was consistent with the color variation. The 1-d group possessed the highest total chlorophyll content (1.4843 mg/g), which corresponded to the most negative a* value and the darkest green in color parameters. It illustrated that ice-crystal formation during freeze–thaw disrupts chloroplast membranes and releases protein-bound chlorophyll into the extraction medium, increasing the extractability [43], further enhancing the greenness of freeze-dried samples. Among hot-air-dried groups, the blanching group exhibited the lowest chlorophyll content (0.1874 mg/g). The total chlorophyll content of the 3-r group (0.2206 mg/g) was comparable to that of the 0-r control group (0.2345 mg/g, no significant difference) and significantly higher than that of the 2-r blanching-only group (0.1874 mg/g). Combined with the color parameter data showing that the a* value of the 3-r group (0.58) was lower than that of the 2-r group (0.65), the results indicated that blanching with green preservation treatment could inhibit chlorophyll degradation, alleviate reddening during drying, and mitigate color deterioration caused by hot-air drying.

#### 3.5.2. Flavor Changes of *Sedum aizoon* L.

Flavor directly determines the eating experience of dried *Sedum aizoon* L. products. In this study, electronic nose technology was applied to objectively analyze the variations in flavor compounds among different treatment groups. Combined with sensory evaluation, quantitative scoring was carried out for characteristic flavors, including grassy and earthy notes, so as to comprehensively evaluate the effects of processing treatments on the flavor quality of dried *Sedum aizoon* L.

As illustrated in Figure 4, *Sedum aizoon* L. exhibited more distinct responses to sensors S2, S7 and S9. Sensor S2 is mainly sensitive to aromatic compounds and nitrogen oxides, corresponding to benzaldehyde, phenethyl alcohol and other components contributing floral and woody aromas to *Sedum aizoon* L. [44,45]. Sensor S7 responds to inorganic sulfides such as hydrogen sulfide, which produce grassy and pungent odors. Sensor S9 is sensitive to organic sulfides including dioctadecyl sulfide [46], which are responsible for fresh vegetable and earthy notes.

The overall sensor response intensities of freeze-dried groups were higher than those of hot-air-dried groups, with notably stronger signals on the three aforementioned sensors. This phenomenon occurred because the high temperature of hot-air drying accelerated the decomposition and loss of partial flavor substances, resulting in reduced flavor diversity and converged flavor profiles among different pretreatment groups. Distinct flavor differences were observed among samples with different pretreatments under the two drying methods. For freeze-dried samples, the sensor responses of S2, S6, S7 and S9 in the blanching group were slightly higher than those of the untreated group, while the opposite trend was found in hot-air dried samples. The blanching combined with green preservation group exhibited the maximum response values of S2, S7 and S9 among all freeze-dried treatments, which significantly promoted the release of aromatic compounds, sulfides, alcohols and aldehydes. Nevertheless, its sensor response intensities were lower than other groups after hot-air drying. The above results demonstrated that the flavor regulation effect of blanching with green preservation was greatly temperature-dependent, and this treatment performed better under low-temperature drying conditions. In summary, drying method was the dominant factor affecting flavor retention of *Sedum aizoon* L., and hot-air drying reduced the flavor diversity of the samples.

In addition, there are certain correlations and deviations between instrumental detection results and human subjective flavor perception. Therefore, sensory evaluation was conducted to analyze the characteristic flavor of samples from five dimensions: grassy taste, bitter taste, sour taste, roasted taste and earthy taste. As shown in Table 8, the roasted flavor scores of hot-air-dried samples (5.07–7.47) were significantly higher than those of vacuum-freeze-dried samples (1.56–2.93). Meanwhile, most hot-air-dried groups obtained higher scores for grassy and earthy flavors. On the contrary, hot-air-dried samples (2.67–4.87) generally exhibited lower bitterness scores than vacuum-freeze-dried samples (4.93–7.23), which could be explained by the thermal degradation of natural bitter compounds such as flavonoids and alkaloids under high temperature. In addition, compared with the control group, freeze–thaw and blanching treatments under hot-air drying enhanced the roasted and earthy flavors of *Sedum aizoon* L. Meanwhile, these pretreatments reduced grassy flavor and bitterness. Notably, the blanching–green preservation group (Group 3-r) obtained significantly lower scores for grassy flavor and bitterness than the control group, which was consistent with the weakened responses of electronic nose sensors S2 and S7. Comprehensive sensory scores reveal that although hot-air drying reduces the flavor diversity of *Sedum aizoon* L., combinations of various pretreatments and hot-air drying can significantly strengthen the roasted and earthy notes of dried products. Meanwhile, undesirable flavors such as astringent grassy taste and bitterness are effectively alleviated. Accordingly, the sensory quality is improved to better meet consumers’ flavor preferences.

In conclusion, hot-air drying reduced volatile diversity but improved overall sensory acceptability by decreasing grassy and bitter notes and enhancing roasted aroma.

### 3.6. Principal Component Analysis

Principal component analysis (PCA) was performed on different processing combinations based on bulk density, tapped density, total phenols, total flavonoids, chlorophyll, VC, total polysaccharides, antioxidant activity, color parameters and flavor properties of *Sedum aizoon* L. As shown in Table 9, four principal components with eigenvalues greater than 1 exhibited a cumulative variance contribution rate of 94.78%, which could explain most information of the original data (≥90%). The result indicated that the quality indicators of *Sedum aizoon* L. could be summarized into four independent principal component factors that sufficiently reflect core differences among indicators. PC1 accounted for 52.86% of the total variance and had a significantly negative correlation with grassy flavor; thus, total polyphenol content was selected as its core indicator. PC2 contributed 18.801%, and bulk density (0.936) showed a strong positive loading, serving as the core indicator of PC2 with weak correlations to other indicators (absolute loading values ≤ 0.206). PC3 contributed 12.563%, and total polysaccharides (0.943) exhibited a strong positive loading with low correlation to other indicators, which was identified as the core indicator of PC3. PC4 accounted for 10.552%, and the absolute loading values of all indicators for PC4 were ≤0.223, without any strongly correlated core indicator. Accordingly, total polyphenols, bulk density and total polysaccharides were finally selected as core indicators. This indicator system covers the physical properties, nutritional quality and functional quality of *Sedum aizoon* L., and can effectively support research on how processing technologies affect its quality characteristics.

### 3.7. Formula Design of Meal Replacement Powder

#### 3.7.1. Analysis of Single-Factor Experiment Results

In this study, *Sedum aizoon* L. powder, skim milk powder, sugar powder, cereal powder and nut powder were used as raw materials of meal replacement powder to investigate the effects of different raw material dosages on sensory scores. As presented in Figure 5, the sensory scores of samples with *Sedum aizoon* L. powder, skim milk powder and sugar powder first increased and then decreased with the rising addition levels, and their optimal dosage ranges were 1~3 g, 2~3 g and 1~2 g, respectively. In contrast, the dosage variations of cereal powder and nut powder exerted slight influences on sensory scores. The maximum sensory score was achieved when the addition amount of cereal powder was 4 g and nut powder was 2.6 g.

#### 3.7.2. Results and Analysis of Factor Screening by Plackett–Burman Design

On the basis of single-factor experiments, a 5-factor, 2-level Plackett–Burman design was adopted in this study, and the experimental results and corresponding analysis are presented in Table 10 and Table 11. The addition amounts of *Sedum aizoon* L. powder and skim milk powder exerted significant effects on sensory scores (*p* < 0.05), while the addition amount of sugar powder showed an extremely significant effect (*p* < 0.01). Therefore, *Sedum aizoon* L. powder, skim milk powder and sugar powder were selected for further response surface optimization and analysis based on the sensory scores of meal replacement powder.

#### 3.7.3. Results of Box–Behnken Design Experiments

The factor levels and results of response surface experiments are listed in Table 12 and Table 13. Multiple regression fitting was performed on the data in Table 12 using Design-Expert software 13.0, with the sensory score of low-GI *Sedum aizoon* L. meal replacement powder as the response variable. The quadratic regression equation was obtained as follows: *Y* = 69.36 + 3.47 *A* + 1.5 *B* + 1.18 *C* − 1.58 *AB* − 1.02 *AC* − 0.225 *BC* − 3.64 *A*^2^ − 0.8925 *B*^2^ − 2.19 *C*^2^. The analysis of variance (ANOVA) for the regression model is shown in Table 14. The *p*-value of the regression model for low-GI *Sedum aizoon* L. meal replacement powder was less than 0.0001, indicating an extremely high significance of the model. Meanwhile, the lack-of-fit term had a *p*-value greater than 0.05, revealing minor experimental interference. The coefficient of determination R^2^ of the model reached 0.9752, reflecting satisfactory fitting performance. The adjusted coefficient of determination R^2^_Adj_ = 0.9432, which meant the model could explain 94.32% of the variation in response values. Therefore, this model can be applied to analyze and predict the sensory scores of the meal replacement powder. According to the F-values, the influences of the three factors on the sensory score of meal replacement powder ranked in descending order as follows: addition amount of *Sedum aizoon* L. powder (A) > addition amount of skim milk powder (B) > addition amount of sugar powder (C). In addition, the interaction term AB had *p* < 0.05, demonstrating that the interaction between the dosages of *Sedum aizoon* L. powder and skim milk powder exerted a significant effect on the sensory score of meal replacement powder. The quadratic terms A^2^ and C^2^ both had *p* < 0.001, which had extremely significant impacts on the sensory score.

#### 3.7.4. Verification Experiment

Design-Expert software predicted that the maximum sensory score of 70.438 could be achieved when the addition amounts of *Sedum aizoon* L. powder, skim milk powder and sugar powder were 2.343 g, 2.759 g and 1.580 g, respectively. Considering practical production requirements, the formula was adjusted to 2.3 g *Sedum aizoon* L. powder, 2.8 g skim milk powder, 1.6 g sugar powder, 4 g cereal powder and 2.6 g nut powder. The actual sensory score was 70.85 ± 1.95, which was basically consistent with the predicted value. The meal replacement powder prepared with this formula exhibited uniform color and balanced flavor, which could effectively mask the sour and astringent taste of *Sedum aizoon* L., and possessed a compound flavor featuring nutty aroma, milk fragrance and mild sweetness. Nevertheless, owing to the different physical properties of raw materials in the meal replacement powder, stratification occurs after mixing with hot water, which impairs the taste and rehydration property of the product. Accordingly, konjac powder needs to be supplemented to improve the viscosity and alleviate stratification.

#### 3.7.5. Determination of Konjac Powder Addition Level

Konjac powder is rich in glucomannan. Its molecular chains form a three-dimensional network structure during hydration. Through hydrogen bonding, hydrophobic interaction and electrostatic interaction, a viscous colloidal solution is formed, which slows down the sedimentation rate of particles in the solution [47,48]. As shown in Figure 6, the sensory score of meal replacement powder increased first and then decreased with the rising addition level of konjac powder, while the viscosity continuously grew. At konjac powder dosages of 0–2%, the sensory scores were relatively low and obvious stratification was observed in the meal replacement powder. When the dosage ranged from 4% to 6%, the sensory scores rose and the product exhibited moderate viscosity. Once the dosage reached 8–10%, the sensory scores declined and the mixture nearly solidified. Consequently, konjac powder with a mass fraction of 4% was selected as the optimal addition amount.

### 3.8. Quality Evaluation of Meal Replacement Powder

The nutritional indicators of the meal replacement powder are listed in Table 14. The dietary fiber content reached 43.4 g/100 g, which is approximately 7.2-fold higher than the minimum threshold of 6 g/100 g required for a “high fiber” nutrition claim under GB 28050–2025 [49] General Rules for Nutrition Labeling of Prepackaged Foods, the product is classified as a high-dietary-fiber food, which can significantly enhance satiety and reduce food intake. Meanwhile, the estimated glycemic index (eGI) is 40.46, well below the low-GI threshold of 55, which can effectively slow down the postprandial blood glucose rise, making it suitable for people with blood sugar control and weight management demands. However, the protein (5.25 g/100 g) and fat (5.87 g/100 g) contents are relatively low; If used as a sole meal replacement, supplementation with a high-quality protein source (e.g., whey or soy protein isolate) to reach approximately 15–20 g protein per serving is recommended to meet adult per-meal protein requirements. If used as a partial meal replacement or snack, it can be consumed alongside regular meals.

To further evaluate the reconstitution properties, the developed product was compared with a commercially available vegetable meal replacement powder (containing kale powder, oat flakes, and flaxseed) (Table 15). The dispersion time (15.02 ± 1.36 s) and wetting time (56.48 ± 2.19 s) of the *Sedum aizoon* L. product were longer than those of the commercial product (4.60 ± 0.64 s and 7.75 ± 0.43 s, respectively), indicating a relatively slower initial wetting and dispersion rate. This may be attributed to the high glucomannan content of konjac powder, which increases inter-particle adhesion upon contact with water. Conversely, the water absorption capacity (751.36 ± 1.41%) and water solubility (47.55 ± 2.69%) of the developed product were higher than those of the commercial reference (702.34 ± 18.48% and 20.87 ± 0.46%, respectively), reflecting the synergistic water-holding effect of *Sedum aizoon* L. powder and konjac powder and the favorable retention of water-soluble polysaccharides after the optimized drying process. The slower wetting and dispersion of the developed product compared with the commercial reference is a trade-off of its high konjac and fiber content. From a product-development perspective, this slower hydration could be reframed as a “gradual thickening” or “sustained-release” feature that prolongs satiety, rather than solely as a drawback. Nevertheless, for consumers seeking instant beverages, granulation or lecithin coating could be explored in future work to improve wettability without compromising fiber content.

To objectively analyze the flavor and taste characteristics, electronic nose and electronic tongue technologies were adopted to quantitatively characterize volatile flavor compounds and non-volatile taste substances, with the commercial vegetable meal replacement powder as a control. As illustrated in Figure 7a, significant differences were observed in the response values of electronic nose sensors 2, 6, 7 and 9 between the two products. The commercial product presented stronger responses on channels corresponding to methane, sulfide, and hydrogen sulfide odors, probably because kale—its major vegetable ingredient—contributes intense grassy and pungent notes. As shown in the electronic tongue profile (Figure 7b), obvious differences were found in astringency, bitterness, sourness, and saltiness. The commercial sample exhibited significantly higher responses in bitterness, astringency, and aftertaste-astringency, and lower responses in saltiness and sourness, compared with the *Sedum aizoon* L. product. This indicated that the synergistic effect of skim milk powder, nut powder, and sugar powder in the formula effectively masked the inherent bitter and astringent taste of the raw materials, delivering a milder and richer taste profile.

Overall, the developed product is a high-fiber, low-GI food with milder flavor and taste profiles than the commercial reference.

## 4. Conclusions

This study systematically evaluated the regulation rules of different pretreatments and drying processes on the quality of *Sedum aizoon* L. Compared with freeze drying, hot air drying delivers higher processing efficiency and better powder flowability, and the hot-air-dried group with blanching for green retention achieves the optimal powder quality. Freeze drying is more conducive to the retention of polyphenols, flavonoids, vitamin C and chlorophyll. The freeze–thaw freeze-dried group has the highest chlorophyll content and the strongest antioxidant activity. Hot air drying combined with pretreatment can effectively reduce grassy odor and bitter taste while enhancing roasted aroma. Single-factor experiments, Plackett–Burman design and Box–Behnken response surface methodology were adopted to establish a well-fitted regression model, and the optimal compound formula of *Sedum aizoon* L.-based meal replacement powder was obtained. Konjac powder was supplemented to eliminate the stratification defect during reconstitution. The finished meal replacement powder is a high-fiber and low-GI food, and its flavor and taste are superior to similar commercial meal replacement products. Per serving, the formula was 2.3 g *Sedum aizoon* L. powder, 2.8 g skim milk powder, 1.6 g sugar powder, 4 g grain powder (oat:quinoa:black highland barley = 3:3:2), 2.6 g nut powder, and 4% konjac powder. The product had an eGI of 40.46 and 43.4 g/100 g dietary fiber. This study fills the research gaps in the processing characteristics of *Sedum aizoon* L. and the development of low-GI meal replacement powder from characteristic green leafy vegetables, and provides innovative ideas and technical support for the large-scale and standardized development of the deep processing industry of *Sedum aizoon* L.

Nevertheless, several limitations remain, including the small size of the trained sensory panel, the in vitro nature of the eGI estimation, and the lack of shelf-life and cost analyses. Future work should incorporate consumer acceptance testing, clinical glycemic validation, and techno-economic assessment under pilot-scale conditions to support commercialization.

## Figures and Tables

**Figure 1:**
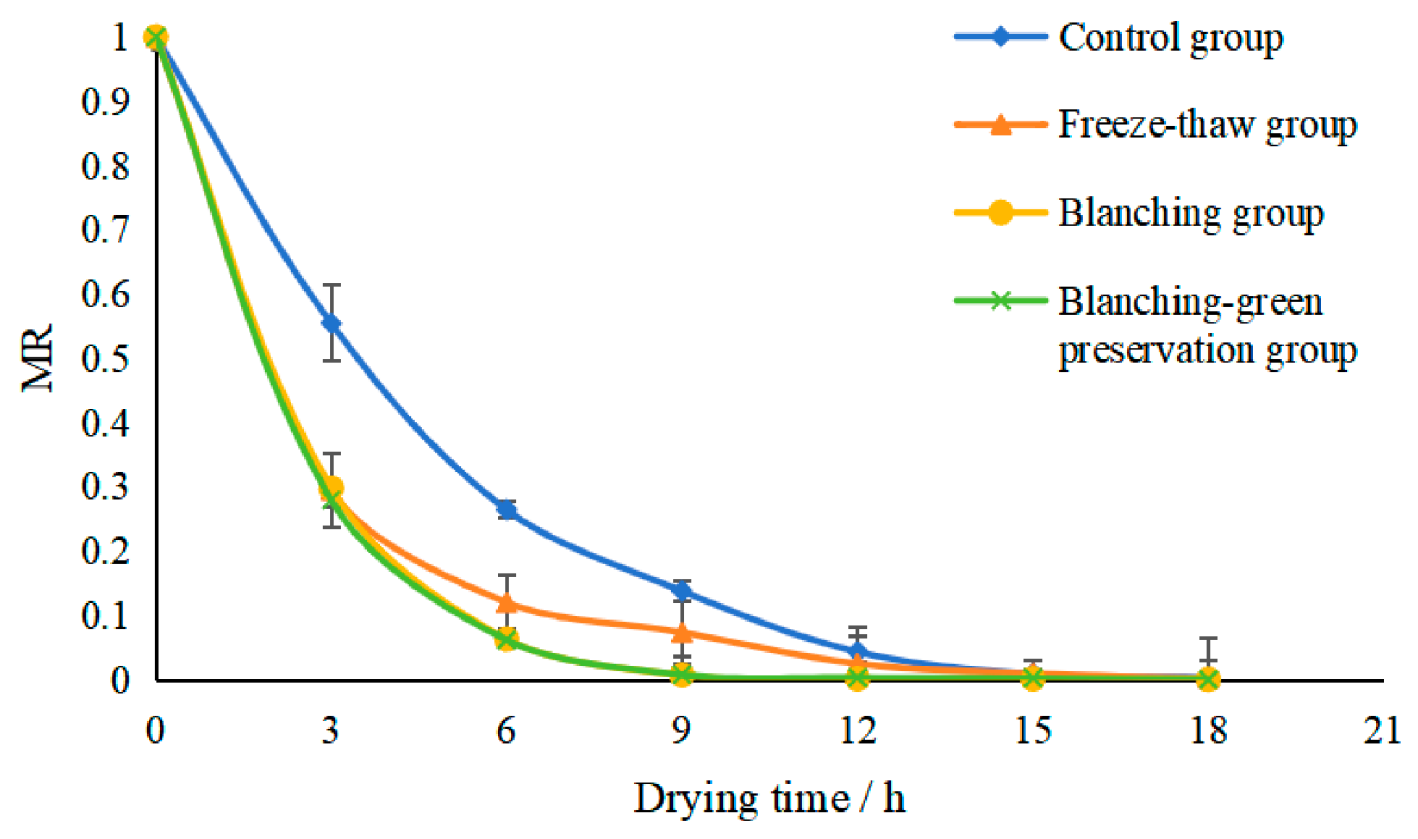
This is a figure. Schemes follow the same formatting.

**Figure 2:**
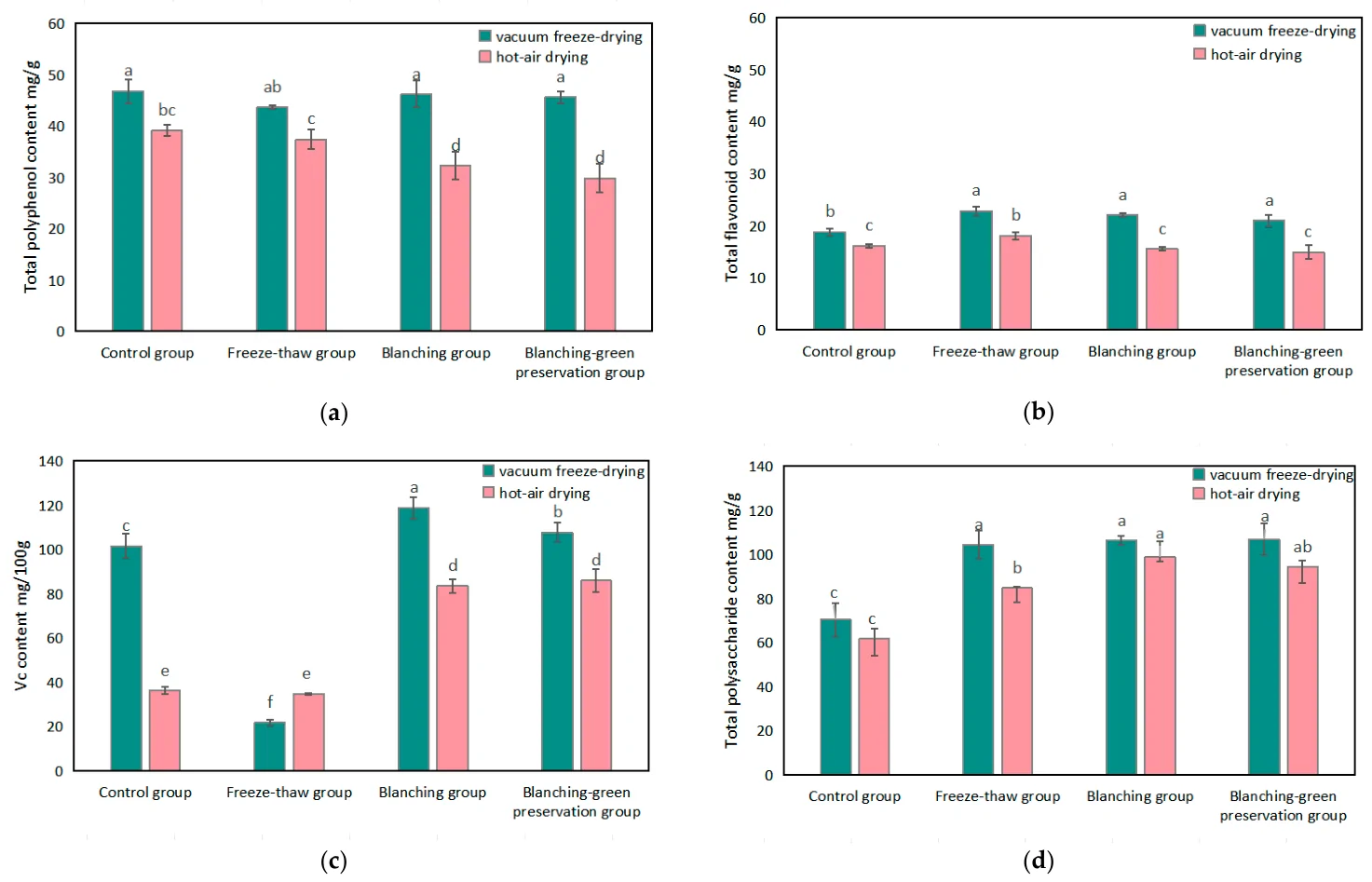
The effects of different processing combinations on the total polyphenols (**a**), total flavonoids (**b**), total polysaccharides (**c**), and VC (**d**) content. Note: Different lowercase letters above the bars indicate significant differences among groups (*p* < 0.05); bars sharing the same letter show no significant difference (*p* ≥ 0.05). *Sedum aizoon* L. is rich in vitamin C (VC), a highly heat-sensitive water-soluble nutrient whose content is vulnerable to processing technologies. Figure 2d presents the VC contents of *Sedum aizoon* L. under different processing combinations. It can be observed that the blanching group and blanching–green preservation group possessed significantly higher VC contents than the untreated group, mainly because blanching shortened the drying duration and effectively alleviated VC degradation. On the contrary, severe VC loss occurred in the freeze–thaw group, whose VC content was markedly lower than that of other treatment groups. Within the freeze–thaw group, samples subjected to hot-air drying retained more VC than vacuum-freeze-dried samples. This could be attributed to cell rupture and juice exudation induced by freeze–thaw treatment, which enlarged the contact area between VC and oxygen and consequently accelerated oxidative degradation.

**Figure 3:**
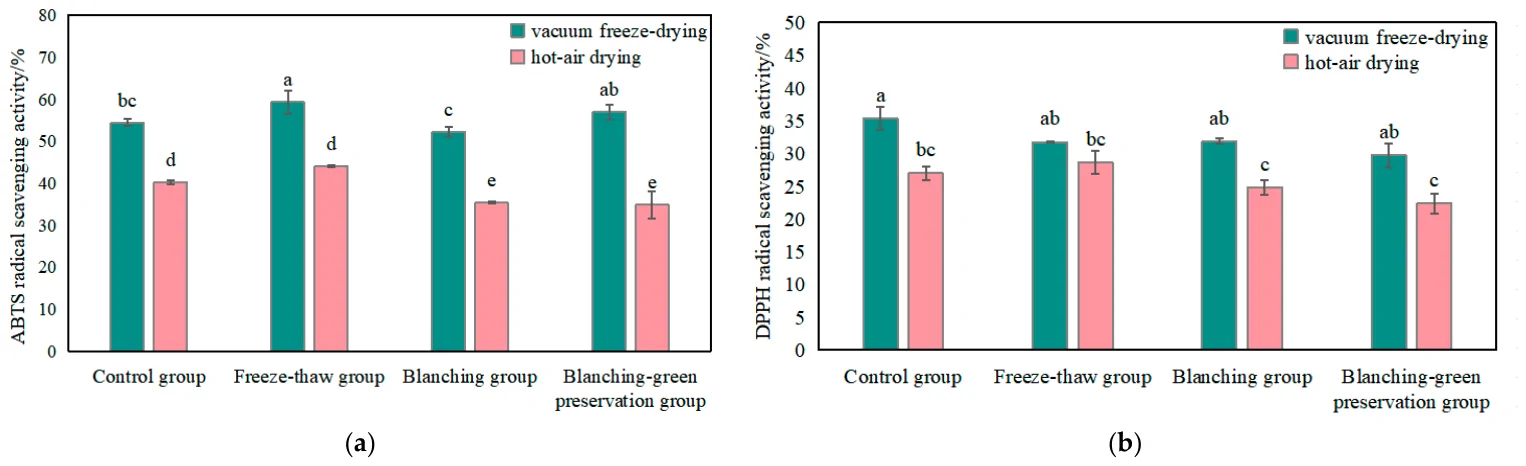
The impact of different processing combinations on antioxidant activity: (**a**) ABTS radical scavenging activity; (**b**) DPPH radical scavenging activity. Note: Different lowercase letters above the bars indicate significant differences among groups (*p* < 0.05); bars sharing the same letter show no significant difference (*p* ≥ 0.05).

**Figure 4:**
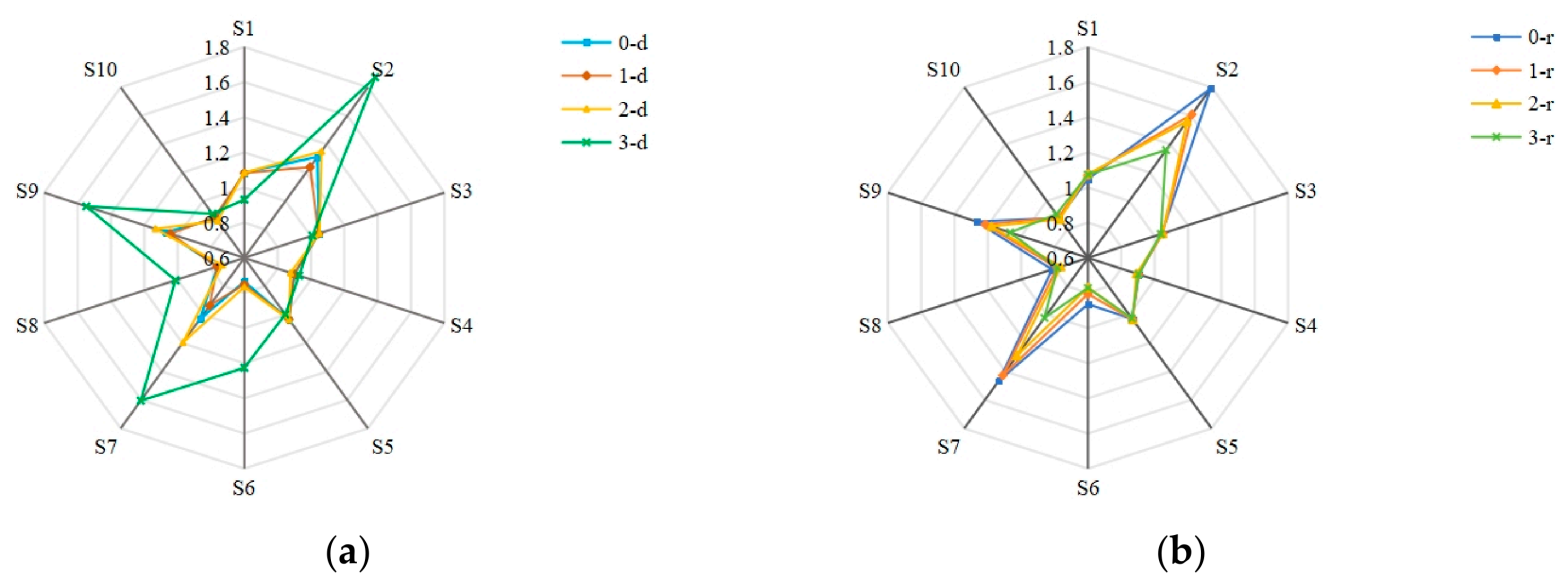
Flavor radar charts of different pretreatments of *Sedum aizoon* L. in freeze drying (**a**) and hot-air drying (**b**).

**Figure 5:**
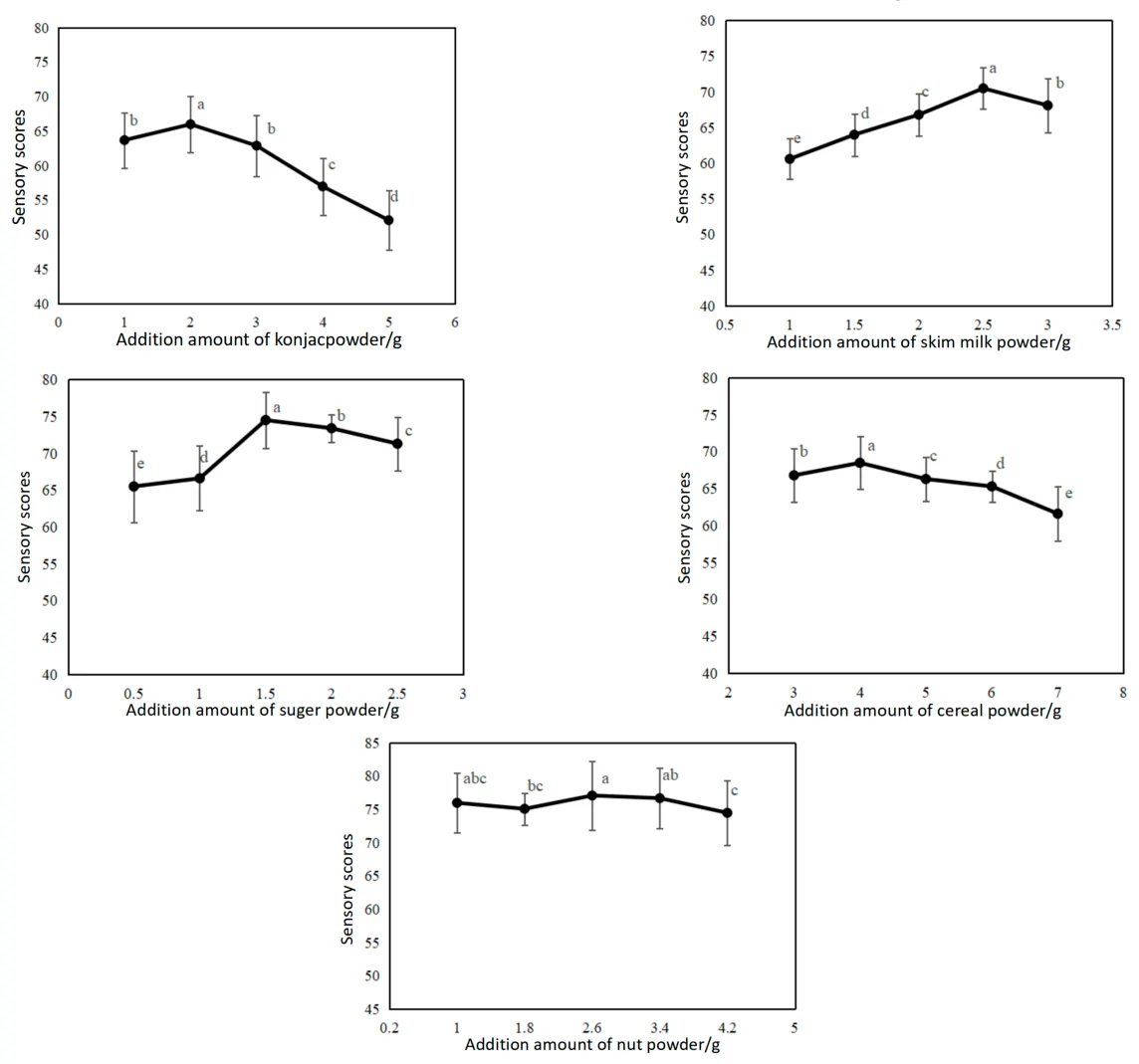
Single-factor experiment results. Note: Different lowercase letters above the data points indicate significant differences among groups (*p* < 0.05); points sharing the same letter show no significant difference (*p* ≥ 0.05).

**Figure 6:**
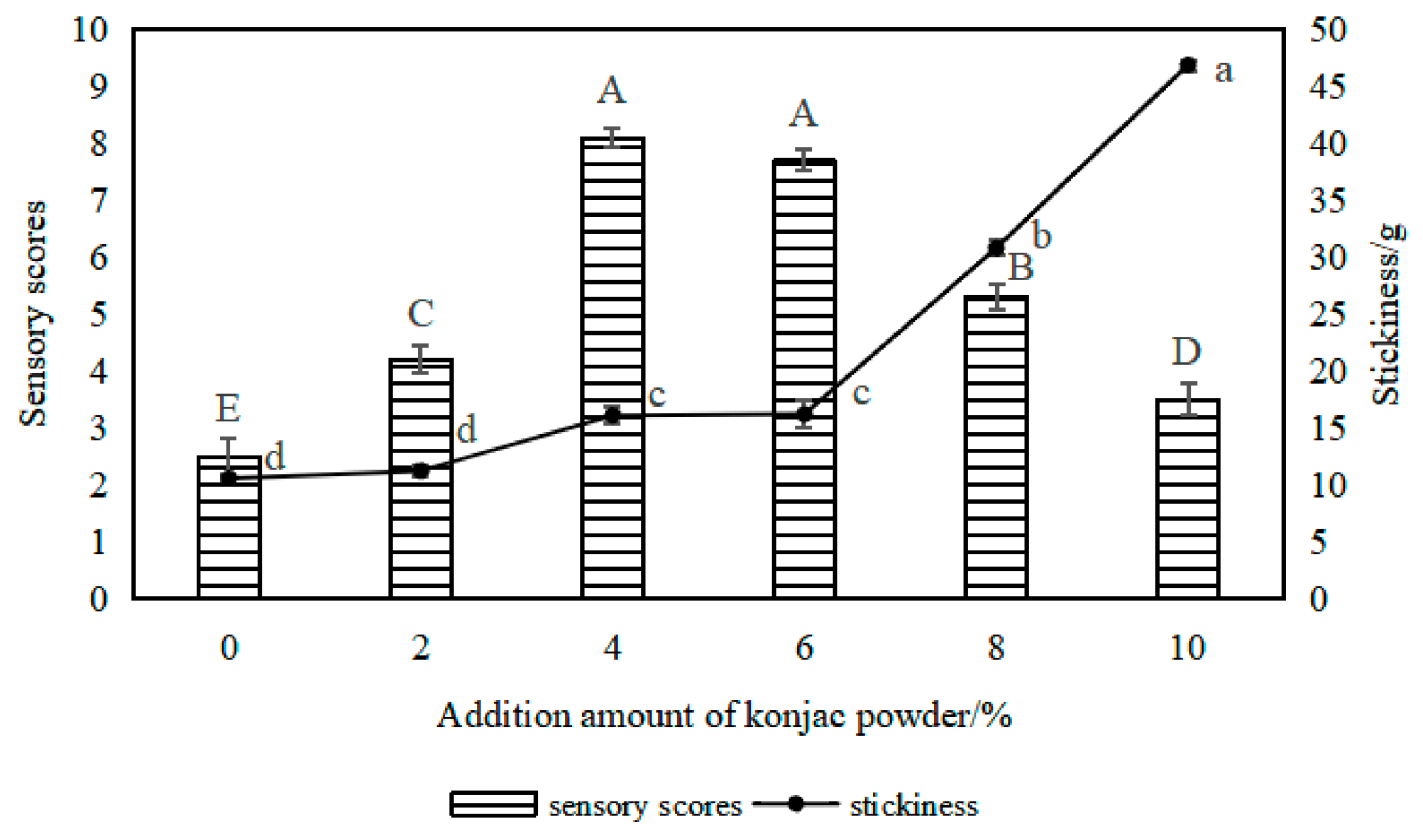
The influence of konjac flour addition level on the sensory score and viscosity. Note: Capital letters above the bars indicate significant differences in sensory scores among groups (*p* < 0.05); lowercase letters next to the data points indicate significant differences in stickiness among groups (*p* < 0.05). Same letters indicate no significant difference (*p* ≥ 0.05).

**Figure 7:**
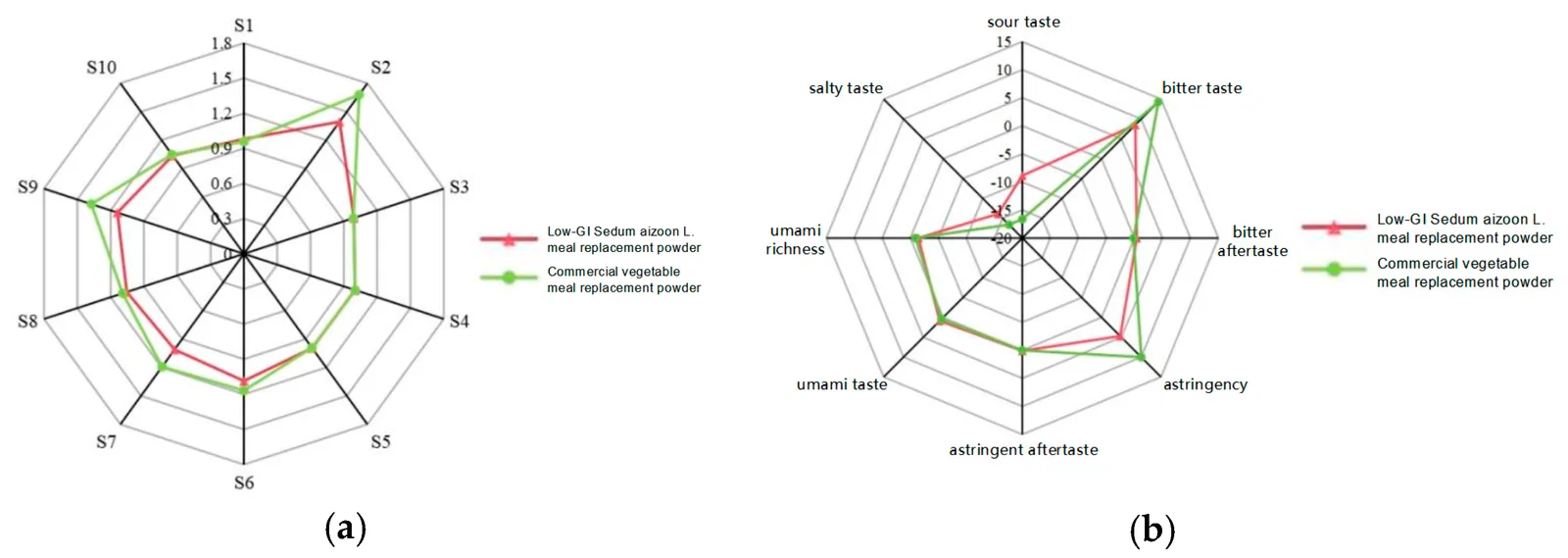
(**a**) Flavor radar chart of meal replacement powder. (**b**) Taste radar chart of meal replacement powder.

**Table 1:** Different sensors of the electronic nose correspond to different types of substances.

Serial No.	Sensor Name	Response Substance Type
S1	W1C	Aromatic compounds
S2	W5S	Nitrogen oxides
S3	W3C	Ammonia compounds and aromatic components
S4	W6S	Hydrides
S5	W5C	Short-chain alkanes and aromatic components
S6	W1S	Alkanes
S7	W1W	Inorganic sulfides
S8	W2S	Alcohols, aldehydes and ketones
S9	W2W	Organic sulfides
S10	W3S	Long-chain alkanes

**Table 2:** Sensory scoring criteria for meal replacement powder.

Item	Scoring Criteria	Sensory Scores
Color (full score: 20 points)	Uniform grass-green color	15–20
	Uneven, too dark or pale	10–14
	Obvious mottling, abnormal color	≤9
Flavor (full score: 30 points)	Balanced mild aroma, no off-flavor	20–30
	Weak/strong but acceptable, no off-flavor	10–19
	Unbalanced, unacceptable flavor	≤9
Taste and mouthfeel (full score: 30 points)	Sweet tasty, smooth texture	20–30
	Mild/strong taste, slight graininess	10–19
	Obvious odd taste, coarse texture	≤9
Reconstitution property (full score: 20 points)	Even texture, proper viscosity, no lumps	15–20
	Even texture, slightly thick/thin, minor lumps	10–14
	Uneven texture, poor viscosity, many lumps	≤9

**Table 3:** Plackett–Burman experimental factor level table.

Factor	Vegetable Powder A	Skim Milk Powder B	Sugar Powder C	Grain Powder D	Nut Powder E
1	1	2	1	3	1.8
−1	3	3	2	5	3.4

**Table 4:** Response surface experiment factor level table.

Factor	Vegetable Powder A	Skim Milk Powder B	Sugar Powder C	Grain Powder D	Nut Powder E
1	1	2	1	1	1
0	2	2.5	1.5	0	2
−1	3	3	2	−1	3

**Table 5:** The influence of different processing combinations on the powder characteristics of *Sedum aizoon* L.

Treatment Group	Bulk Density (g/cm^3^)	Tapped Density (g/cm^3^)	Hausner Ratio	Carr Index (%)
0-r	0.34 ± 0.02 ^b^	0.59 ± 0.02 ^a^	1.75 ± 0.03 ^b^	42.77 ± 0.94 ^b^
0-d	0.16 ± 0.02 ^d^	0.23 ± 0.03 ^cd^	1.46 ± 0.00 ^d^	31.64 ± 0.09 ^d^
1-r	0.19 ± 0.02 ^c^	0.28 ± 0.03 ^c^	1.47 ± 0.02 ^d^	32.09 ± 0.80 ^d^
1-d	0.07 ± 0.00 ^f^	0.15 ± 0.01 ^e^	1.94 ± 0.08 ^a^	48.42 ± 2.24 ^a^
2-r	0.36 ± 0.01 ^b^	0.49 ± 0.05 ^b^	1.35 ± 0.08 ^ef^	25.67 ± 4.36 ^e^
2-d	0.11 ± 0.00 ^e^	0.18 ± 0.04 ^de^	1.58 ± 0.01 ^c^	36.87 ± 0.04 ^c^
3-r	0.43 ± 0.01 ^a^	0.55 ± 0.01 ^a^	1.26 ± 0.03 ^f^	20.46 ± 2.00 ^f^
3-d	0.11 ± 0.01 ^e^	0.15 ± 0.01 ^e^	1.39 ± 0.01 ^de^	27.92 ± 0.41 ^de^

Notes: 0-r represents the treatment group with no pretreatment followed by hot-air drying; 0-d represents the treatment group with no pretreatment followed by freeze drying; 1-r represents the freeze–thaw pretreatment + hot-air-drying group; 1-d represents the freeze–thaw pretreatment + freeze-drying group; 2-r represents the blanching pretreatment + hot-air-drying group; 2-d represents the blanching pretreatment + freeze-drying group; 3-r represents the blanching with green preservation pretreatment + hot-air-drying group; 3-d represents the blanching with green preservation pretreatment + freeze-drying group. Values in the same column followed by different lowercase letters are significantly different (*p* < 0.05), while those marked with the same letter show no significant difference (*p* ≥ 0.05). The same applies to the following tables.

**Table 6:** Color change of *Sedum aizoon* L.

Treatment Group	L*	a*	b*
0-r	52.36 ± 4.70 ^c^	0.22 ± 0.09 ^a^	12.16 ± 2.54 ^ab^
0-d	54.53 ± 1.88 ^b^	−3.67 ± 0.34 ^b^	9.32 ± 0.81 ^bc^
1-r	50.20 ± 1.96 ^cd^	0.85 ± 0.20 ^a^	7.54 ± 1.69 ^d^
1-d	61.99 ± 1.00 ^a^	−5.00 ± 0.66 ^c^	14.10 ± 2.22 ^a^
2-r	42.99 ± 3.24 ^e^	0.65 ± 0.25 ^a^	3.22 ± 1.03 ^e^
2-d	59.08 ± 1.37 ^a^	−4.14 ± 1.43 ^bc^	12.33 ± 1.66 ^ab^
3-r	47.15 ± 1.75 ^de^	0.58 ± 0.11 ^a^	10.58 ± 2.48 ^bc^
3-d	60.40 ± 1.34 ^a^	−4.43 ± 0.61 ^bc^	14.85 ± 0.64 ^a^

Notes: Values in the same column followed by different lowercase letters are sig-nificantly different (*p* < 0.05), while those marked with the same letter show no signifi-cant difference (*p* ≥ 0.05).

**Table 7:** Changes in chlorophyll content of *Sedum aizoon* L.

Treatment Group	Chlorophyll A Content (mg/g)	Chlorophyll B Content (mg/g)	Total Chlorophyll Content (mg/g)
0-r	0.0854 ± 0.0046 ^f^	0.1502 ± 0.0076 ^f^	0.2345 ± 0.0122 ^ef^
0-d	0.2840 ± 0.0039 ^d^	0.5101 ± 0.0050 ^d^	0.7967 ± 0.0090 ^d^
1-r	0.1138 ± 0.0022 ^e^	0.2059 ± 0.0090 ^e^	0.3215 ± 0.0112 ^e^
1-d	0.5178 ± 0.0191 ^a^	0.9533 ± 0.0338 ^a^	1.4843 ± 0.0530 ^a^
2-r	0.0613 ± 0.0068 ^f^	0.1211 ± 0.0131 ^g^	0.1874 ± 0.0200 ^f^
2-d	0.4318 ± 0.0092 ^b^	0.7575 ± 0.0204 ^b^	1.1826 ± 0.0294 ^b^
3-r	0.0797 ± 0.0019 ^f^	0.1439 ± 0.0023 ^fg^	0.2206 ± 0.0042 ^ef^
3-d	0.3468 ± 0.0016 ^c^	0.6301 ± 0.0060 ^c^	0.9778 ± 0.0076 ^c^

Notes: Values in the same column followed by different lowercase letters are sig-nificantly different (*p* < 0.05), while those marked with the same letter show no signifi-cant difference (*p* ≥ 0.05).

**Table 8:** Sensory scores of different combination processes.

Treatment Group	Grassy Odor	Roasted Aroma	Bitterness	Sourness	Earthy Odor
0-r	7.13 ± 0.74 ^b^	5.07 ± 0.70 ^d^	2.13 ± 0.52 ^a^	4.87 ± 0.72 ^c^	5.77 ± 0.73 ^d^
0-d	8.17 ± 0.67 ^a^	1.56 ± 0.65 ^h^	2.13 ± 0.67 ^a^	5.94 ± 1.03 ^b^	5.13 ± 0.85 ^f^
1-r	4.70 ± 0.87 ^e^	6.97 ± 0.79 ^c^	1.37 ± 0.44 ^c^	2.67 ± 0.72 ^f^	7.17 ± 0.79 ^a^
1-d	8.16 ± 0.79 ^a^	1.63 ± 0.88 ^g^	1.57 ± 0.49 ^b^	7.23 ± 0.82 ^a^	5.43 ± 0.73 ^e^
2-r	5.07 ± 0.70 ^d^	7.60 ± 0.91 ^a^	1.23 ± 0.70 ^cd^	4.20 ± 0.68 ^d^	6.20 ± 0.59 ^c^
2-d	7.00 ± 0.68 ^c^	2.87 ± 0.64 ^f^	1.13 ± 0.44 ^d^	4.93 ± 0.82 ^c^	3.93 ± 0.70 ^h^
3-r	4.13 ± 0.83 ^f^	7.47 ± 0.64 ^b^	1.20 ± 0.41 ^cd^	3.40 ± 0.74 ^e^	6.41 ± 0.67 ^b^
3-d	7.07 ± 0.70 ^c^	2.93 ± 0.73 ^e^	1.27 ± 0.42 ^cd^	5.00 ± 0.82 ^c^	4.27 ± 0.86 ^g^

Notes: Values in the same column followed by different lowercase letters are sig-nificantly different (*p* < 0.05), while those marked with the same letter show no significant difference (*p* ≥ 0.05).

**Table 9:** Principal component loading matrix, eigenvalues, and contribution rates.

Properties	Principal Component			
	PC1	PC2	PC3	PC4
Bulk density	−0.11	0.936	0.218	−0.214
Tapped density	0.933	−0.067	0.251	0.210
Total polyphenols	0.934	0.206	−0.117	−0.223
Total flavonoids	0.661	0.200	−0.648	0.168
Total polysaccharides	0.234	−0.41	0.943	0.215
Grassy odor	−0.961	0.095	0.102	0.034
Eigenvalue	8.458	3.008	2.010	1.688
Contribution rate/%	52.863	18.801	12.563	10.552
Cumulative contribution rate/%	52.863	71.663	84.226	94.778

**Table 10:** Plackett–Burman experimental design and results.

Number	Addition Amount/g					
	A *Sedum aizoon* L. Powder	B Skim Milk Powder	C Sugar Powder	D Grain Powder	E Nut Powder	Sensory Score
1	1	−1	1	1	1	67.5
2	1	−1	−1	−1	1	51.9
3	1	−1	1	1	−1	66.7
4	1	1	−1	1	1	60.0
5	−1	−1	1	−1	1	62.0
6	−1	1	−1	1	1	64.0
7	−1	−1	−1	−1	−1	59.5
8	−1	−1	−1	1	−1	57.1
9	−1	1	1	1	−1	77.6
10	1	1	−1	−1	−1	56
11	−1	1	1	−1	1	74
12	1	1	1	−1	−1	65

**Table 11:** Variance analysis of Plackett–Burman test results.

Item	SUM of Squares	df	Mean Square	F-Value	*p*-Value	Significance
Model	541.08	5	108.22	11.02	0.0055	
A	61.20	1	61.20	6.23	0.0468	*
B	84.80	1	84.80	8.63	0.0260	*
C	344.54	1	344.54	35.07	0.0010	**
D	50.02	1	50.02	5.09	0.0649	
E	0.5208	1	0.5209	0.0530	0.8255	
Residual	58.94	6	9.82			
Total	600.03	11				

Note: * indicates significant difference compared with the control group (*p* < 0.05); ** indicates highly significant difference (*p* < 0.01).

**Table 12:** Response surface experimental design and results.

Number	A *Sedum aizoon* L. Powder/g	B Skim Milk Powder/g	C Sugar Powder/g	Sensory Score
1	1	3	1.5	63.8
2	2	2.5	1.5	69.9
3	2	2	2	66.2
4	3	2.5	1	66.6
5	3	2.5	2	66.5
6	2	2.5	1.5	69.2
7	2	3	2	69.1
8	2	2.5	1.5	69
9	1	2.5	2	62.5
10	1	2.5	1	58.5
11	2	2	1	63
12	1	2	1.5	58
13	3	2	1.5	69
14	2	2.5	1.5	70.6
15	2	2.5	1.5	68.1
16	3	3	1.5	68.5
17	2	3	1	66.8

**Table 13:** Analysis of variance for quadratic model.

Item	Sum of Squares	df	Mean Square	F-Value	*p*-Value	Significance
Model	226.11	9	25.12	30.55	<0.0001	**
A	96.60	1	96.60	117.46	<0.0001	**
B	18.00	1	18.00	21.89	0.0023	**
C	11.04	1	11.04	13.43	0.0080	**
AB	9.92	1	9.92	12.06	0.0104	*
AC	4.20	1	4.20	5.11	0.0583	
BC	0.2025	1	0.2025	0.2462	0.6349	
A^2^	55.86	1	55.86	67.93	<0.0001	**
B^2^	3.351	1	3.351	4.08	0.0832	
C^2^	20.24	1	20.24	24.61	0.0016	**
Residual	5.76	7	0.8224			
Lack of fit	2.18	3	0.7283	0.8156	0.5486	
Pure error	3.57	4	0.8930			
Total variation	231.87	16				
R^2^ = 0.9752			R^2^_Adj_ = 0.9432			

Note: * indicates significant difference compared with the control group (*p* < 0.05); ** indicates highly significant difference (*p* < 0.01).

**Table 14:** Nutritional indicators of meal replacement powder.

Index	Content	Index	Content
Moisture	5.66 g/100 g	Starch	35.96 g/100 g
Protein	5.25 g/100 g	eGI	40.46
Fat	5.87 g/100 g	Dietary fiber	43.43 g/100 g

Note: The eGI value was estimated using an in vitro enzymatic digestion method; actual human glycemic responses may vary.

**Table 15:** Characteristic indicators of meal replacement powder.

Index	Developed Product	Commercial Product
Dispersibility (s)	15.02 ± 1.36	4.60 ± 0.64
Wettability (s)	56.48 ± 2.19	7.75 ± 0.43
Water absorption capacity (%)	751.36 ± 1.41	702.34 ± 18.48
Water solubility (%)	47.55 ± 2.69	20.87 ± 0.46

## Data Availability

The original contributions presented in this study are included in the article/Supplementary Materials. Further inquiries can be directed to the corresponding author.

## Supplementary Materials

The following supporting information can be downloaded at: https://www.mdpi.com/article/10.3390/foods15193379/s1, Table S1: Qualitative analysis of active components in *Sedum aizoon* L.
