# Supplementary material for: Research on Processing Characteristics of Sedum aizoon L. and Development of Low-GI Meal Replacement Powder
Source: Foods. 2026 Sep 23;15(19):3379. doi: 10.3390/foods15193379 (PMC13664476; doi:10.3390/foods15193379)
Supplement: Supplementary file 1 [file foods-15-03379-s001.zip › foods-4504429-supplementary.pdf]

Table S1. Qualitative analysis of active components in *Sedum aizoon L.*

Bioactive category	Compound					
	**Positive ion mode**	**Response value**	**Molecular formula**	**Negative ion mode**	**Response value**	**Molecular formula**
Flavonoids	Epigallocatechin gallate	54586	$C_{22}H_{18}O_{11}$	Quercetin-3-O-glucoside	435934	$C_{21}H_{20}O_{12}$
	Rutin	58360	$C_{27}H_{30}O_{16}$	Myricitrin	435934	$C_{21}H_{20}O_{12}$
	Myricetin	222375	$C_{15}H_{10}O_8$	Kaempferol-3-O-α-L-rhamnoside	82646	$C_{21}H_{20}O_{10}$
	Quercetin	148996	$C_{15}H_{10}O_7$	Quercetin	73770	$C_{15}H_{10}O_7$
	Rhamnetin-3-O-glucoside	7762	$C_{22}H_{22}O_{12}$	Luteolin	253498	$C_{15}H_{10}O_6$
	Luteolin	114607	$C_{15}H_{10}O_6$	Scutellarein	253498	$C_{15}H_{10}O_6$
	Kaempferol	8267	$C_{16}H_{12}O_7$	Isorhamnetin	83502	$C_{16}H_{12}O_7$
Phenolic acids				p-Hydroxybenzoic acid	2440	$C_7H_6O_3$
				Protocatechuic acid	2440	C7H6O4
				Caffeic acid	47639	$C_9H_8O_4$
				p-Coumaric acid	9912	$C_9H_8O_3$
Triterpenoids	Shionone	38273	$C_{30}H_{50}O$	Ursolic acid	3587	$C_{30}H_{48}O_3$
				Oleanolic acid	3587	$C_{30}H_{48}O_3$
Fatty acids	Methyl linoleate	25697	$C_{19}H_{34}O_2$	Linoleic acid	104927	$C_{18}H_{32}O_2$
Vitamins	Vitamin C	32200	$C_6H_8O_6$	Vitamin C	1016756	$C_6H_8O_6$
	Vitamin K_1	27580	$C_{31}H_{46}O_2$			
Organic acids				Malic acid	389918	$C_4H_6O_5$
Esters				Dimethyl phthalate	435934	$C_{10}H_{10}O_4$
Anthraquinones				Chrysophanol-8-O-β-D-glucoside	9040	$C_{21}H_{20}O_9$
